# Putative Association between Low Baseline Gene Expression in the Peripheral Blood and Clinical Remission in Rheumatoid Arthritis Patients Treated with Tofacitinib

**DOI:** 10.3390/life11121385

**Published:** 2021-12-11

**Authors:** Elena V. Tchetina, Azamat M. Satybaldyev, Galina A. Markova, Elena Yu. Samarkina, Aleksandr M. Lila

**Affiliations:** 1 Immunology and Molecular Biology Laboratory, Nasonova Research Institute of Rheumatology, 115522 Moscow, Russia; g.markova2010@yandex.ru (G.A.M.); samarkinale@list.ru (E.Y.S.); 2 Early Rheumatoid Arthritis Department, Nasonova Research Institute of Rheumatology, 115522 Moscow, Russia; azamatsat@yandex.ru (A.M.S.); amlila@mail.ru (A.M.L.)

**Keywords:** rheumatoid arthritis, tofacitinib, remission, gene expression, whole blood

## Abstract

We investigated the importance of the baseline expression of genes involved in energy generation, as prognostic biomarkers of the treatment response to tofacitinib in patients with rheumatoid arthritis (RA). Peripheral blood samples were obtained from 28 patients with RA who received 3 months of tofacitinib therapy from 26 healthy controls. Clinical response was evaluated based on the disease activity score, the erythrocyte sedimentation rate (DAS28-ESR), and the serum levels of ACPA, RF, CRP, and ESR. Clinical remission was assessed based on DAS28 score <2.6. Protein concentrations were measured using ELISA. Total RNA isolated from whole blood was used for gene expression analysis using quantitative RT-PCR. All patients were diagnosed with Steinbrocker’s radiographic stage II-III at baseline, and most showed erosive arthritis with ACPA and RF positivity. Tofacitinib treatment significantly decreased the disease activity. Upon study completion, seven patients showed remission. Before and after TOFA therapy, a significantly higher expression of succinate dehydrogenase and pyruvate kinase genes was observed in all the examined patients compared to healthy subjects. However, the pre-therapy expression of these genes and corresponding proteins was significantly (*p* ≤ 0.05) lower in patients who showed remission than in other patients with RA. Moreover, we observed that, during follow-up, patients who developed remission showed an increasing trend in the expression of the examined genes, whereas the others showed some decreases in gene expression, although this was not statistically significant. We concluded that, compared with RA patients maintaining persistent moderate or high disease activity, those with clinical remission following tofacitinib treatment showed a significantly lower baseline expression of genes involved in energy generation.

## 1. Introduction

Rheumatoid arthritis (RA), an autoimmune rheumatic disease of unknown aetiology is characterised by chronic erosive arthritis and systemic inflammatory lesions involving various internal organs. The prevalence of RA among adults is 0.5–2%. RA affects all age groups, including children and elderly populations, with the highest prevalence among those aged 30–55 years. RA is characterised by a wide heterogeneity of clinical manifestations owing to various combinations of disease phenotypes, which include varying degrees of joint impairment that may range from damage caused by minor cartilage injury to significant bone erosions and disparities in the disease course and outcomes [1].

Currently, autoimmune processes and inflammatory cascades, mainly those associated with the tumour necrosis factor (TNF)α-signaling pathway are therapeutic targets in RA, that aim to alleviate symptoms [2]. However, occasionally, the ineffectiveness of these therapeutic agent limits the long-term prescription of drugs for patients with RA [3], which necessitates therapy modification. Although many patients show an adequate response to therapy, 40% of patients with RA are refractory to methotrexate (MTX) as a first-line treatment, which needs to be switched to a biological disease-modifying anti-rheumatic drug (DMARD) regimen [4]. However, an additional 40% of patients with RA are unresponsive to the second-line drug; therefore, approximately 20% of patients require additional therapy [5].

Previous studies reported that the systemic inflammation observed in RA is associated with metabolic changes secondary to disturbances in the energy production in cells of various tissues [6]. In many cells and tissues, energy in the form of ATP is generated during glucose oxidation in glycolysis involving the activity of two ATP-producing enzymes: phosphoglycerate kinase and pyruvate kinase (PKM). Glucose breakdown in glycolysis yields pyruvate, which is decarboxylated to generate acetyl-CoA; then, this is further oxidised to carbon dioxide and water in the citric acid cycle (CAC). The reactions of the cycle are performed by enzymes that generate reduced equivalents of nicotinamide adenine dinucleotide (NADH) and flavin adenine dinucleotide (FADH2). These reduced equivalents proceed through oxidative phosphorylation in the respiratory electron transport chain during ATP generation. Succinate dehydrogenase is the only enzyme involved in both the CAC and the electron transport chain (respiratory complex II) [7].

RA T cells differ from those in healthy age-matched subjects, primarily by the abnormal expression of metabolic regulators [8] and premature aging associated with functional decline [9]. This is also associated with changes in cellular metabolism resulting in attenuated ATP synthesis by mitochondria and the provision of CAC intermediates [10]. The mitochondrial dysfunction observed in RA T cells results in increased ROS production [11]. ROS facilitate the dimerisation of the cytosolic enzyme, PKM2, and its nuclear translocation, where it activates STAT3 [12].

The Janus kinase (JAK)-signal transducer and activator of the transcription (STAT) signalling pathway is implicated as a critical pathogenic contributor to RA [13]. The JAK/STAT pathway is a key mediator of pro-inflammatory pathways that operates in autoimmune diseases, since it is activated by several cytokines, such as interferons, interleukin (IL)-2, and IL-6, which control the viability, proliferation, and differentiation of various types of cells [14].

Tofacitinib (TOFA) is a first-generation JAK1/3 inhibitor that is structurally similar to ATP. It acts as a reversible, competitive inhibitor of ATP in the ATP-binding site of the active carboxyterminal protein kinase domain (JH1) of JAK proteins, promoting their inactivation followed by the STAT protein blockade [15]. Tofacitinib is a DMARD used for monotherapy in patients with RA. Treatment outcomes following TOFA therapy are significantly better than those associated with methotrexate in the treatment of naive patients with RA and those with an inadequate response to biological DMARDs [16]. Recent studies showed that TOFA reduced reactive oxygen species production and regulated the expression of key mitochondrial genes, increased oxidative phosphorylation and ATP production in synovial tissue explants obtained from patients with RA accompanied by a reduced glycolytic activity [17].

However, the evaluation of joint tissue for treatment prognosis is unacceptable during the early stages of the disease, and drawing blood samples is technically easier and less painful for patients. Therefore, in this study, we used peripheral blood mononuclear cells (PBMCs) obtained from patients with RA. Moreover, T cells in patients with RA showed high energy requirements [18]; therefore, we deduced that both sensitivity and resistance to TOFA therapy may be associated with disturbances in the energy metabolism of these vital components of the immune system. Here, in our preliminary study, we hypothesised that the expression of genes involved in energy-generating metabolic pathways: glycolysis (PKM2) and oxidative phosphorylation (succinate dehydrogenase subunit B (SDHB)) measured in the peripheral blood of TOFA-naive patients with RA prior to TOFA therapy may serve as useful biomarkers to identify patients who may show remission.

## 2. Materials and Methods

### 2.1. Ethics Statement

Our study was performed in accordance with the Declaration of Helsinki. The study protocol (No. 13 dated 4 June 2015) was approved by the Local Human Research Ethics Committee, and informed consent was obtained from all subjects.

### 2.2. Patients

The study included 28 patients with RA (6 men and 22 women) aged ≥ 20 years (median age 55 [39.5; 62] years) who did not receive tofacitinib previously. All patients were diagnosed based on the American College of Rheumatology (ACR) classification criteria.

TOFA was prescribed to patients owing to refractoriness (unsatisfactory efficacy) to previous treatment: 25 of 28 patients were treated with methotrexate (at a dose of 20–25 mg/week), methotrexate as a combination therapy with methylprednisolone (8 mg per day) (in 4 of 28 patients), and biological therapy (in 9 of 28 patients). One patient was treated with adalimumab (40 mg subcutaneously once every 2 weeks) in combination with leflunomide (20 mg); one patient with methylprednisolone and tocilizumab; and another patient was not given any treatment.

All patients received TOFA (5–10 mg twice a day) during 3 months of follow-up. Among them, 23 of 28 patients with RA received methotrexate (20–25 mg/week), in addition to TOFA; 4 of 28 patients received TOFA and continued to receive methylprednisolone (8 mg/day) (2 patients received methylprednisolone + MTX + TOFA; 1 patient, methylprednisolone + hydroxychloroquine (0.2 mg/day) +TOFA; and 1 patient, TOFA + methylprednisolone). One patient received adalimumab (40 mg subcutaneously once every 2 weeks) + MTX + TOFA. Another patient received leflunomide (20 mg daily) + TOFA. Two patients received TOFA only.

All patients were evaluated by the same rheumatologist during follow-up. The following were the inclusion criteria for this study: (a) Confirmed diagnosis of RA (based on the ACR-European League Against Rheumatism 2010 or ACR 1987 criteria). (b) Medium and high disease activity (DAS28 > 3.2). (c) Aged between 20 and 80 years. (d) Availability of written informed consent. (e) Refractoriness and/or intolerance of previous therapy. (f) Availability of adequate contraceptive measures in patients of childbearing potential. The following were exclusion criteria: (a) Pregnancy and lactation. (b) Severe active infections, organ dysfunctions, or hematological disorders. (c) Demyelinating diseases. (d) Malignancies or precancerous conditions. (e) Alcohol or drug addiction. (f) A history of allergic reactions to protein drugs. (g) Immunisation with live and attenuated vaccines 4 weeks before study enrollment. Remission was defined using the ACR criteria for clinical remission based on the simplified 28-joint score (DAS28). The control group included of 26 subjects (7 men, 19 women; median age, 52 [41.5; 59.5] years; range 19–69 years) without current, chronic or acute infections and no family history of autoimmune diseases.

### 2.3. Demographic, Clinical, and Immunological Evaluations

The following data were obtained at baseline and at 3 months: disease duration, Steinbrocker’s radiographic stage, and the DAS28-ESR score. Patients’ age and sex were also indicated. Nephelometric analysis was performed using a BN-100 analyzer (Dade Bering, Germany) to measure serum C-reactive protein (CRP, cut-off value 5 mg/L) and immunoglobulin M rheumatoid factor (RF, a standard cut-off value 15 mU/L) concentrations. Anti-citrullinated protein autoantibodies (ACPA) were measured using enzyme-linked immunosorbent assay (ELISA) kit according to the manufacturer’s recommendations (cut-off level 5 U/mL for antibody positivity) (Axis Shield Diagnostics Limited, Dundee, UK).

### 2.4. Quantification of SDHB and PKM2 Protein Levels

SDHB (SEJ756Hu) and PKM2(SEA588Hu) concentrations were measured in isolated PBMCs using commercially available ELISA kits (Cloud-Clone Corp, Houston, TX, USA) according to the manufacturer’s instructions. PBMC lysates were obtained using Cell Extraction Buffer containing 10 mM Tris, pH 7.4, 100 mM NaCl, 1 mM EDTA, 1 mM EGTA, 1 mM NaF, 20 mM Na_4_P_2_O_7_, 20 mM Na_3_VO_4_, 1% Triton X-100, 10% glycerol, 0.1% SDS, and 0.5% deoxycholate (Invitrogen, Camarillo, CA, USA), supplemented with a Protease Inhibitor Cocktail (Sigma-Aldrich, Inc., St. Louis, MO, USA) and 1 mM PMSF (Sigma-Aldrich, Inc., St. Louis, MO, USA) according to the manufacturer’s instructions. The total DNA content in PBMC lysates was measured spectrophotometrically using GeneQuant (Amersham Biosciences, Piscataway, NJ, USA). The results are expressed per µg of DNA.

### 2.5. Total RNA Isolation and Reverse Transcriptase (RT) Reaction

Total RNA was isolated from 100 µL of whole blood immediately after withdrawal using a Ribo-zol-A kit. The RT-reaction was performed using a Reverta kit that contains M-MLV Reverse Transcriptase, random hexanucleotide primers and total RNA, according to the manufacturer’s recommendations (InterLabService, Moscow, Russia).

### 2.6. Real-Time Quantitative Polymerase Chain Reaction (PCR)

The following pre-made primers and probes were used to perform the TaqMan assay (Applied Biosystems, Foster City, CA, USA): PKM2 (Hs00987255_m1) and SDHB (Hs01042482_m1). β-Actin was used as an endogenous control. Gene expression was quantified using the Quant Studio 5 Real-Time PCR System (Applied Biosystems, Foster City, CA, USA) as described previously [19]. Briefly, 1 μL of RT product was subjected to real-time PCR in a total reaction mixture (15 μL) containing 7.5 μL of TaqMan Universal PCR Master Mix (Applied Biosystems), 900 nM sense and antisense primers, 50 nM probe, and template cDNA. After a single step at 50 °C for 2 min and initial activation at 95 °C for 10 min, the reaction mixtures were subjected to 40 amplification cycles (15 s at 95 °C for denaturation and 1 min of annealing and extension at 60 °C). Relative mRNA expression was determined using the ΔΔC_T_ method per the manufacturer’s guidelines (Applied Biosystems). The ΔC_T_ value was calculated by subtracting the C_T_ value for the housekeeping gene from the C_T_ value for each sample. Subsequently, the ΔΔC_T_ value was calculated by subtracting the ΔC_T_ value of each control (each healthy subject) from the ΔC_T_ value observed in each patient with RA.

### 2.7. Statistical Analysis

Quantitative data are expressed as medians (25th and 75th percentiles). All statistical analyses were performed using the Statistica software package (version 12.0 StatSoft) and were performed in duplicate. The Mann–Whitney and Wilcoxon signed-rank tests were used for statistical processing of the results. Pearson’s correlation analysis was used for normally distributed data. A *p* value ≤ 0.05 was considered statistically significant. All statistically significant differences are indicated with an asterisk (*).

## 3. Results

### 3.1. Clinical and Immunological Characteristics and Therapeutic Response in Patients with RA at Baseline and after 3 Months of Tofacitinib Therapy

The mean duration of RA in the examined patients was 24 months, which varied from 4 to 156 months. At baseline, twenty-one patients presented with Steinbrocker’s radiographic stage II disease and seven patients had stage III RA. Three patients were seronegative for ACCP, and four were negative for RF, the remaining (25 and 24 patients, respectively) were seropositive for both biomarkers. At the beginning of the study, 15 patients with RA had a high disease activity (DAS28 > 5.1) (three patients from subgroup 1 and 12, from subgroup 2) and 13 had a moderate activity (3.2 < DAS28 < 5.1) (four patients from subgroup 1 and 9 from subgroup 2). However, after the 3-month follow-up, we observed a significant decrease in disease activity based on DAS28 scores (*p* < 0.001), serum CRP levels (*p* = 0.007), and the number of swollen (*p* < 0.001) and tender joints (*p* < 0.001). Upon completion, a high disease activity persisted in 4 patients (all of them belonged to subgroup 2), moderate disease activity was observed in 17 patients (all of them were from subgroup 2), and remission was observed in 7 (25%, DAS28 < 2.6): three patients initially showed a high disease activity and four presented with moderate disease activity. Of the 17 patients who showed a moderate disease activity, eight initially showed a high activity, and moderate disease activity persisted in nine patients. As we sought to obtain biomarkers for remission attainment, all of the patients were divided into two subgroups according to DAS28 indices at the end of the study. Subgroup 1 consisted of seven patients with RA who developed remission after three months of tofacitinib treatment while subgroup 2 patients (*n* = 21) maintained high and moderate RA activity at the end of the study.

We observed no significant differences in baseline characteristics between patients who achieved remission (subgroup 1) and other patients with RA (subgroup 2). We observed a trend of a higher number of tender joints in patients in subgroup 2. Upon study completion, patients from both subgroups showed a significant decrease in disease activity (subgroup 1, *p* = 0.01; subgroup 2, *p* < 0.001) and in the number of swollen (subgroup 2, *p* = 0.003) and tender (subgroup 2, *p* < 0.001) joints (Table 1). Patients who achieved remission showed a decrease in serum CRP levels and ESR, whereas patients from subgroup 2 showed increased ESR.

### 3.2. Whole-Blood Gene Expression Profiles in Patients with Rheumatoid Arthritis at Baseline and after 3 Months Tofacitinib Therapy

**Figure 1:**
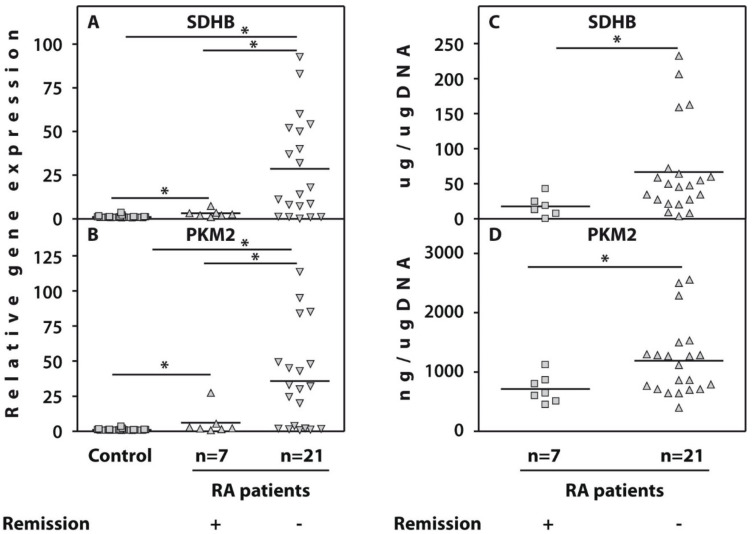
Baseline relative expression of the genes’ succinate dehydrogenase (SDHB, (**A**)) and pyruvate kinase (PKM2, (**B**)) with reference to 𝛽-actin as determined using real-time PCR analyses in whole blood from patients with RA who achieved remission (*n* = 7) (Remission +) or other examined patients with RA (*n* = 21) (Remission −) compared to healthy controls (*n* = 26). The controls are shown as 1.0 as required for relative quantification with the real-time PCR protocol. Protein concentrations of SDHB (**C**) and PKM2 (**D**) measured by ELISA in PBMCs from same subgroups of RA patients. Asterisks (*) indicate significant differences between the examined subgroups of patients (Mann–Whitney U-test).

Gene expression analysis of peripheral blood in patients with RA pre- and post-TOFA therapy revealed a significantly higher expression of the SDHB and PKM2 genes in both subgroups of patients than in healthy subjects both before and after TOFA treatment. However, the pre-therapy expression of these genes was significantly (*p* ≤ 0.05) lower in patients who showed remission than in other patients with RA (Figure 1A,B). Moreover, we observed that, during follow-up, patients who developed remission showed a trend of an increased expression of the examined genes (*p* = 0.06 in the case of SDHB, and *p* = 0.15 in the case of PKM2), whereas the others showed a minor decrease in gene expression, although this was not statistically significant (Table 1).

### 3.3. Succinate Dehydrogenase Subunit B and Protein Kinase Protein Expression in Isolated Peripheral Blood Mononuclear Cells

We analysed SDHB and PKM2 concentrations in the PBMC fraction to further investigate the clinical significance of SDHB and PKM2 relative gene expression in the peripheral blood of patients with RA; we observed that at baseline, SDHB and PKM2 protein expressions in PBMCs were significantly lower in patients with RA who achieved remission than in other patients with RA (Figure 1C,D).

### 3.4. Correlation between Gene Expression and Clinical Parameters

Comparisons of Pearson’s correlation coefficients for baseline expression of SDHB and PKM2 genes in relation to each other in the entire study population comprising patients with RA (*n* = 28) revealed a strong correlation (r = 0.544, *p* = 0.003). Bivariate correlation analyses, using Pearson’s correlation coefficients for baseline expressions of the aforementioned genes with the clinical parameters, revealed a negative correlation between PKM2 gene expression and the ΔDAS28 after 3 months of follow-up (r = −0.433, *p* = 0.02) and only a nonsignificant trend with regard to SDHB expression (r= −0.274, *p* = 0.17). We also observed a positive correlation between baseline PKM2 gene expression and the number of tender joints before treatment (r = 0.392, *p* = 0.04) and DAS28 scores after 3 months follow-up (r = 0.508, *p* = 0.008).

## 4. Discussion

In this preliminary study, we observed that the post-TOFA therapy improvement in patients with RA was associated with the baseline status of energy-producing metabolic pathways. Specifically, patients who achieved clinical remission according to DAS28-ESR score showed a significantly lower baseline expression of PKM2 and SDHB genes, which are associated with ATP production in glycolysis and the Krebs cycle, respectively. This finding was supported by the results of correlation analyses, which revealed a negative correlation between these gene expressions and the ΔDAS28 values. Our results concur with the findings of a previous study that reported an association between the TOFA-induced inhibition of the JAK-STAT pathway and alterations in cellular mitochondrial function [17], as well as the finding that the activation of AMP-activated protein kinase (a major regulator of cellular ATP concentration) can inhibit JAK-STAT-dependent pro-inflammatory signalling [20]. Moreover, our findings can be considered in a clinical setting for the assessment of the possibility of clinical remission development in patients with RA prior to tofacitinib therapy.

Mechanistically, the putative association of tofacitinib effect and cellular energy metabolic conversions could be caused by the ‘super-effector cell’ functions of RA T cells with a low threshold for effector cytokine production [18], which is mediated by the activation of JAK proteins [15]. Moreover, cytokine protein synthesis is a high-energy-consuming process [21]. Therefore, it is conceivable that the expressions of enzymes related to ATP production, such as PKM2 and SDHB, were upregulated in all the examined patients with RA prior to therapy.

Being structurally similar to ATP, tofacitinib inhibits the JAK-STAT pathway, thus limiting inflammation and joint destruction in both of the examined patient subgroups. However, the inhibition of the JAK-STAT pathway in subgroup 1 patients in the course of clinical remission development was associated with a trend of further upregulation in the expression of genes associated with ATP production compared to healthy subjects, which might later promote disease relapse. This observation supports the notion that clinical remission represents some reprogramming of cellular metabolism and its adaptation to new, but still unhealthy conditions, rather than returning to healthy phenotypes [22]. Consequently, patients classified as being in remission according to the DAS28 score are indeed not in a state compatible with true remission [23] and could be undertreated [24], as indicated previously. Therefore, our results highlight an important issue related to the validity of clinical remission assessment criteria in patients with RA treated with tofacitinib. In view of our findings, the assessment of the metabolic status of patients with RA at the gene expression level prior to treatment could serve as an important criterion for therapy administration.

In this study, all patients with RA showed a reduced number of tender joints during the course of follow-up, which is consistent with a finding of a previous study that reported an association of the JAK inhibitor efficacy in monitoring pain caused by both inflammation and other conditions [25]. A previous study that reported the role of glycolysis upregulation in pain sensitivity in patients with RA [26] supports the positive correlation of the baseline PKM2 gene expression and the number of tender joints observed before treatment.

The remission rate in response to tofacitinib after three months of treatment observed in our study (25%), was comparable with that reported previously [27]. On the other hand, although we did not observe significant differences between baseline DAS28 scores in the examined subgroups, the average initial DAS28 value in patients with RA who developed remission after TOFA treatment was lower than that in the other subgroup. In addition, subgroup 2 patients, who demonstrated a higher baseline expression of the examined genes, showed a trend of a higher number of tender joints compared to subgroup 1. These observations might indicate that a higher RA severity in subgroup 2 patients is associated with unresponsiveness to TOFA treatment. The association of a greater baseline disease activity with a reduced likelihood of achieving sustained remission was also noted previously [28].

The present pilot study had several limitations. Due to the relatively small size of the examined cohort and short duration of the follow-up that consequently underpowered the study, there is clearly a need for this analysis to be repeated in a much larger sample with a longer duration of observations, in order to avoid the effect of possible confounders. In addition, we cannot determine whether the therapy-associated increase in the gene expressions of SDHB and PKM2 is specific for tofacitinib. Indeed, our study is the first to use energy-related metabolic genes to predict the treatment response. Therefore, this subject requires further investigation. We believe that this type of response might be more common, as energy-related genes might be involved in metabolic responses to other treatments. Another limitation of our study is related to PBMC heterogeneity, which might result in different gene expression levels of SDHB and PKM2 in different PBMC subsets. Therefore, the assessment of the proportion of T cells, B cells, and monocytes in PBMCs of the examined subjects is interesting and could be significant. However, for the selection of a drug before treatment of a patient with RA, which requires a rapid decision, determining gene expression in the whole PBMC fraction could be a suitable approach.

## 5. Conclusions

Our preliminary study highlights that changes in PKM2 and SDHB gene expressions in the peripheral blood in the TOFA-naïve patients with RA are associated with clinical response to treatment. Therefore, considering the metabolic status of patients with RA, we conclude that the baseline gene expression analysis of peripheral blood may be useful for identifying prognostic biomarkers of responsiveness to TOFA therapy. Further studies are warranted to investigate the energy metabolism disturbances observed in patients with RA in response to therapy to gain a deeper understanding of the internal “drivers” of rheumatic processes, in order to facilitate the development of novel therapeutic approaches to control RA.

## Figures and Tables

**Table 1:** Patient characteristics and relative expression of pyruvate kinase (PKM2) and succinate dehydrogenase subunit B (SDHB) genes in patients who achieved remission (*n* = 7) and other examined patients with RA (*n* = 21), before and after tofacitinib therapy.

	Subgroup 1 Patients Who Achieved Remission (*n* = 7) DAS28 < 2.6		*p*	Subgroup 2 Other Examined Patients with RA (*n* = 21) ΔDAS > 1.2; DAS28 > 2.6		*p*	*p’* Subgroup 1 versus Subgroup 2 Prior to Treatment
	Baseline Median (IQR)	After 3 Months Median (IQR)		Baseline Median (IQR)	After 3 Months Median (IQR)		
Age, years	53 [36; 51]			61.5 [56; 68]		0.42	
Disease duration, months	33 [16; 143.5]			24 [10.5; 54]		0.33	
CRP, mg/ml	15 [4.4; 43]	5.4 [0.25; 10]	0.06	25 [11.4; 42.6]	4.3 [0.9; 12.9]	0.04 *	0.49
ESR, mm	22 [26; 62]	16 [10; 20]	0.06	35 [21; 45]	46 [18; 70]	0.27	0.79
DAS 28	5.04 [4; 5.5]	1.96 [1.68; 2.2]	0.01 *	5.4 [4.7; 6.3]	4.4 [3.3; 5.1]	<0.001 *	0.26
ΔDAS 28		3.08			1.0		
Number of swollen joints	7 [3; 9]	0	-	9 [6; 10,5]	2 [1; 6]	0.003 *	0.3
Number of tender joints	5 [3; 9]	0	-	9 [5.5; 13.5]	2 [1; 9]	<0.001 *	0.07
Gene expression							
SDHB	2.53 [2; 3.5]	9.24 [3.3; 267.5]	0.06	16.0 [4.3; 51]	4.7 [2.6; 21]	0.8	0.04 *
PKM2	2.24 [1.63; 5.15]	18.9 [3.68; 259.2]	0.15	31 [2; 48.6]	13.3 [3.1; 27]	0.7	0.05 *

Asterisks (*) indicate significant differences between the examined subgroups of patients. (P-Wilcoxon signed-rank test; P’- Mann–Whitney U-test). Abbreviations: CRP, C-reactive protein; ESR, erythrocyte sedimentation rate; DAS, disease activity score; SDHB, succinate dehydrogenase; PKM, pyruvate kinase.

## Data Availability

Data supporting reported results can be found at http://quinta.online/RA (accessed on 5 December 2021).
